# Secession

**Published:** 1861-01

**Authors:** 


### Secession.

It seems that Southern Medical Students have again found it neccessary to secede from a Northern School. The University Medical College, N. Y., has been the theatre of a more serious difficulty recently, between Southern Students and the Faculty, than that which occurred at Jefferson Medical College, Philadelphia, twelve months ago. The reports of the affair from the New York secular papers, and the Medical Times of that city, do not give a very satisfactory explanation of the cause of disturbance. It seems that Dr. Aylette, the blind private teacher in New York—perhaps native of Alabama—a great favorite of Southern Medical Students, was attacked by the Faculty, through their President, in the following letter:

"Dr. Aylette:—Dear Sir: Will you please give me replies for the use of the Faculty to the four following question:

"1. Have you informed any student that it is not necessary to take out his tickets at the beginning of the secession, and that the Faculty did not require their fees until Christmas?

"2. Have you taken money from students who had brought it to New York for the purpose of paying their college fees, and invested it, for your own profit, with buisiness men?

"3. Have you, after receiving New York funds, given to any uncurrent notes at a heavy discount, keeping the difference for your own use?

"4. Have you failed to repay any student who had deposited his money for safe keeping, on the excuse that those to whom you had lent it were unable to keep their engagement with you?

"Your eary reply to these questions will greatly oblige

J. W. DRAPER,

*President Medical Faculty, N. Y. U. M. C.*"

After the above letter had been received, Dr. Aylette's students held a meeting and passed the following resolutions:

"*Whereas*, a communication was received by Dr. P. A. Aylette, our associate and friend, from Professor John W. Draper, President of, and in behaf of the Faculty of the Medical Department of the University of New York propounding certain interrogatories, which, in their manner and language, we think, contain imputations against the character of Dr. Aylette, which we know to be unfounded and untrue, and which deservedly meet our unanimous disapprobation, and which, we regret, are calculated to militate seriously against the prosperity of this institution—in proof of which we have only to look at the large majority of students from that section of the country which has been, and will be, induced by the existing state of affairs. Therefore,

"*Resolved*, That in our professional social, and personal association with Dr. P. A. Aylette, we have ever found him the courteous gentleman, the prudent counsellor companion and friend; and that any imputation cast upon his personal integrity is untrue, and we boldly assert that the originator of these charges is guilty of malicious and unprovoked slander.

"*Resolved*, In our opinion, the long-continued and most successful instructions of Dr. Aylette, of which our predecessors and ourselves have been the recipients, in the medical department of this University, have materially contributed to the attainment of its present prosperity.

"*Resolved*, That in our connexion with this institution, during the present and previous sessions, we have always found Dr. Aylette its unflinching friend; and in order to promote its interests has left no efforts untried to prevent students being led astray by the political agitation of the times.. And so far from deriving pecuniaiy profit from the use of their money, has incurred, to our knoweledge, personal loss in affording them accommodation, by the exchange of uncurrent for current funds."

We think some cause existed beyond that given in these reports, which, if disclosed, would make the explanation more satisfactory. Why were these charges made by the Faculty indirectly, in the form of interrogatories, and why were they made at all, without evidence of their correctness? Certainly the suspicion of a stampede by the students, and the return of Dr. Aylette to the South, were not sufficient reasons to the Faculty for the attempt to blast his character; nor would the course pursued be justified upon the ground that no further service could be expected, or that his influence might be exerted in another quarter.

We know nothing of Dr. Aylette, personally, nor of the students who defended him against the charges, but we must say, that it appears to us, from the showing of the friends of the University themselves, that there was an attempt to defame the character of Dr. Aylette, without showing just cause.

